# Myosin Heavy Chain 9 (MYH9)-Related Congenital Macrothrombocytopenia

**DOI:** 10.7759/cureus.16964

**Published:** 2021-08-06

**Authors:** Aswani Thurlapati, Srinandan Guntupalli, Richard Mansour

**Affiliations:** 1 Internal Medicine, Louisiana State University Health Sciences Center, Shreveport, USA; 2 Hematology and Oncology, Louisiana State University Health Sciences Center, Shreveport, USA; 3 Oncology, Louisiana State University Health Sciences Center, Shreveport, USA

**Keywords:** thrombocytopenia, myh-9 related disorder, dohle-body, fechtner syndrome, may-hegglin disorders, myosin heavy chain 9

## Abstract

Myosin heavy chain 9 (MYH9)-related hereditary macrothrombocytopenia is caused by mutation of the MYH9 gene encoding the heavy chain A of non-muscle myosin of class II. We present a case that emphasizes the importance of awareness of rare disorders which could potentially avoid over-investigation, especially in benign conditions.

A 72-year-old Caucasian female presented for preoperative evaluation for cataract extraction. She was noted to have thrombocytopenia of 30 K/uL along with elevated creatinine. She denied any acute symptoms except for a prolonged history of easy bruising. Physical exam revealed bruising over the extremities. Upon further questioning, she was previously investigated for thrombocytopenia and had multiple diagnostic as well as therapeutic interventions including bone marrow biopsies, steroids, intravenous immunoglobulins with no improvement. Her family history is consistent with low platelet counts for at least three generations. Peripheral blood smear showed large platelets, normal red and white blood cells with Döhle bodies. Further genetic testing revealed an inherited MYH9 mutation which is autosomal dominant.

MYH9-related disorders are characterized by macrothrombocytopenia, often associated with glomerulonephritis, sensorineural deafness, cataracts, and cytoplasmic inclusion bodies within leukocytes. Management is mainly conservative and directed towards the prevention of iron deficiency anemia in young females. The use of desmopressin, in combination with tranexamic acid, is recommended in a perioperative setting. Our case emphasizes the importance of history-taking skills that could potentially minimize further diagnostic or therapeutic interventions in this benign genetic disorder.

## Introduction

Causes of thrombocytopenia can range from simple medications to life-threatening microangiopathies. However, at times patients are falsely labeled as immune thrombocytopenic purpura (ITP) after basic workup. Even though it is rare, some patients may have congenital causes of thrombocytopenia. One such group of congenital thrombocytopenia disorders is MYH9-related platelet disorders. With a prevalence of 1-9/100,000 MYH9-related platelet disorders are a rare group of autosomal dominant congenital macrothrombocytopenia. It consists of four distinct disorders, which include May-Hegglin anomaly, Epstein syndrome, Fechtner syndrome, and Sebastian platelet syndrome. Diagnosis is confirmed by testing for mutation of the MYH9 gene. Here we report a patient with ITP, who on further investigation is diagnosed with the Fechtner type of congenital MYH9-related platelet disorder. 

## Case presentation

A 73-year-old female with a past medical history of immune thrombocytopenic purpura (ITP), end-stage renal disease (ESRD), bilateral lens detachment, hypertension, heart failure with reduced ejection fraction was referred to the hematology and oncology clinic for easy bruising and persistent thrombocytopenia. She had no apparent history of epistaxis, bleeding gums, post-menopausal bleeding, melena, hematochezia, hematemesis, fever, chills, or joint pains.

Since her early 20s, the patient was noted to have thrombocytopenia with easy bruising. She has undergone multiple bone marrow biopsies which were unremarkable. She was diagnosed with ITP and started treatment with chronic steroids. However, this has caused unwarranted osteopenia and uncontrolled hyperglycemia and hence was discontinued. The patient was then lost to follow-up with hematologists and had not visited one in more than 25 years. During these years, she started to develop worsening renal function, ultimately leading to ESRD, requiring hemodialysis. She later developed bilateral lens detachment, requiring fixation and replacement. Family history is remarkable for chronic thrombocytopenia in her son and grandson, who were never further evaluated. 

Her medications consisted of amiodarone, carvedilol, valsartan-sacubitril, pantoprazole, sevelamer carbonate, erythropoietin injection during dialysis, sertraline, trazodone, and multivitamin supplements. On physical examination, the patient was thin built. Generalized purpura was noted on the extremities. On cardiac examination, a systolic flow murmur was audible over the mitral region. Labs were significant for the following as mentioned in Table 1 below. 

**Table 1:** Laboratory findings seen in our patient WBC: white blood cells; TIBC: total iron-binding capacity; BUN: blood urea nitrogen; INR: international normalized ratio; PTT: partial thromboplastin time; AST: aspartate aminotransferase; ALT: alanine transaminase

Lab Test	Patient’s Values	Normal Values
WBC	4,000 K/ul	3.9-12.7 K/ul
Platelet Count	30 K/ul	150-450 K/ul
Hemoglobin	11.3 g/dL	10.9-14.1 g/dL
Iron	46 ug/dl	30-160 ug/dl
TIBC	207 ug/dl	265-497 ug/dl
Iron Saturation	22%	20%-50%
Absolute Reticulocyte Count	0.070 M/ul	0.023-0.095 M/ul
BUN	47 mg/DL	8-23 mg/DL
Creatinine	3.9 mg/DL	0.5-1.4 mg/DL
INR	1.04	0.8-1.1
PTT	31 seconds	25-36 seconds
AST	20 U/L	10-40 U/L
ALT	21 U/L	10-44 U/L
Total Bilirubin	0.5 mg/dl	0.1-1.1 mg/dl
Erythropoietin level	77 mIU/mL	2.6-18.5 mIU/mL
Vitamin B12	1101 pg/mL	210-950 pg/mL
Methylmalonic acid	1.19 umol/L	<0.40 umol/L

**Figure 1:**
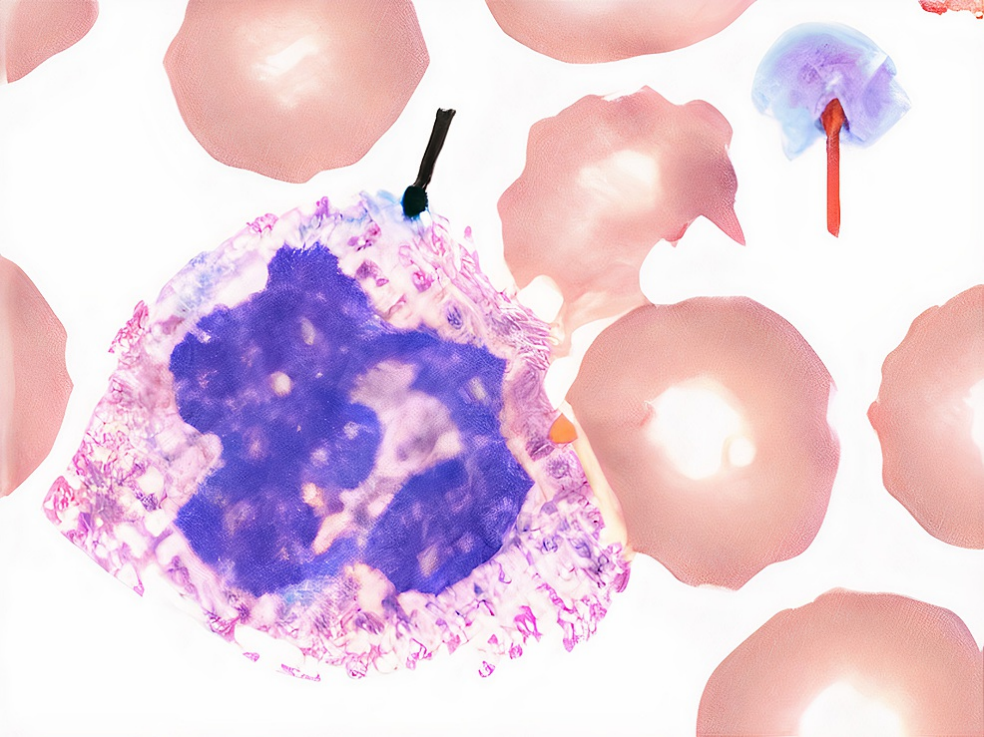
Patient's peripheral smear findings Black arrow - gray-white, cotton wool-like neutrophilic inclusion body; red arrow - large platelet

**Figure 2:**
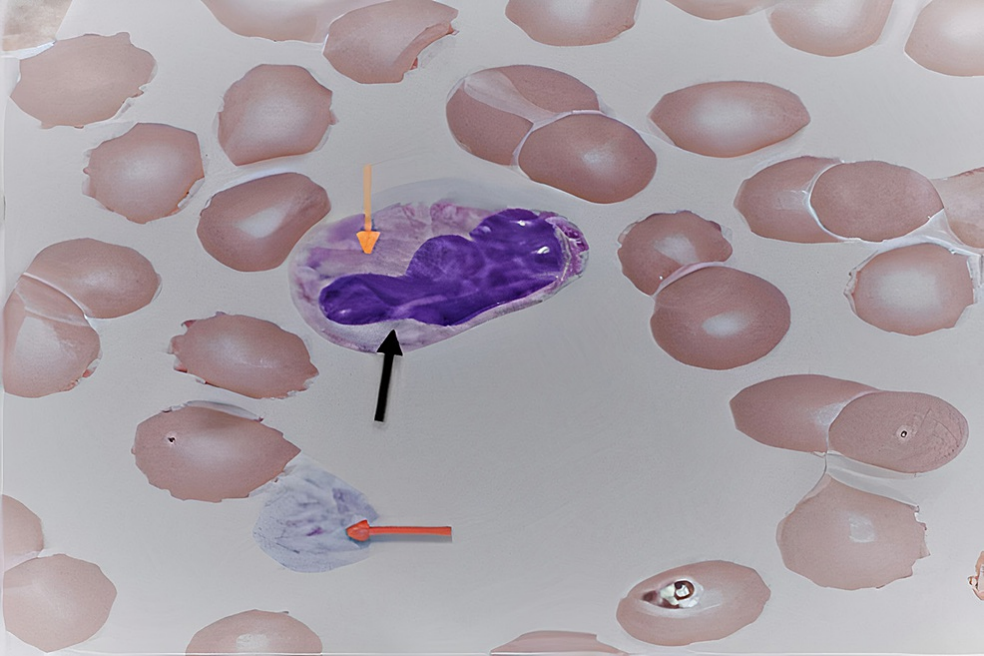
Patient's peripheral smear Black and orange arrows: "Döhle bodies": grey-white cotton-wool like inclusion bodies; Red arrow: large platelet

Peripheral smear revealed a mixture of large and normal-sized platelets with some granulocytes containing grey-cotton wisp inclusion bodies as seen in Figure 1 and Figure 2. 

Due to the characteristic peripheral smear findings, significant family history, and benign serum chemistries, congenital platelet disorders were high on our differentials. This included MYH9-related disorders, Bernard-Soulier syndrome, gray platelet syndrome. Hence, we performed a genetic analysis with next-generation sequencing (NGS), which revealed heterozygous variant mutation of MYH9 gene with C.3493C>T. 

## Discussion

Given our patient's significant family history and clinical spectrum of lens detachment, ESRD, and peripheral smear with inclusion bodies and large platelets, congenital thrombocytopenia was suspected. A genetic analysis was performed which revealed a previously undiagnosed MYH9 related disorder. Studies suggest the use of genotyping should be incorporated at an early stage in the diagnostic pathway for better preventive strategies and outcomes [1]. 

Epidemiology of MYH9 platelet disorders

Dr. May described a family in which several members had enlarged platelets with rare bleeding abnormalities in 1909. Later in 1945, Dr. Hegglin, a swiss physician described Döhle body-like inclusions in the leukocytes of affected individuals with a dominantly inherited giant platelet disorder. This led to the term May-Hegglin anomaly (MHA) to describe the triad of thrombocytopenia, giant platelets, and leukocyte inclusion bodies [2]. With around 300 unrelated families reported in medical literature, MYH9 platelet disorders are a rare group of congenital platelet disorders, which are mainly autosomal dominant in transmission as seen in our patient’s family [3]. However, in 30% of the patients, the mutations could be novel without predisposing family history [4].

Pathophysiology

MYH9 gene is present on chromosome 22q12-13 and encodes the nonmuscle myosin heavy chain class IIA (NMMHC-IIA), a cytoskeletal contractile protein that is part of a family of three variants, which include type A, type B, and type C. They play a significant role in cytokinesis, phagocytosis, cell motility, and cellular membrane integrity [5]. Platelets and leukocytes exclusively contain only the type-A variant, hence an MYH9 gene mutation causes enlarged and fragile platelets, which easily get destroyed. There is also a clumping of the nonfunctional myosin-9 protein to aggregate, forming the characteristic inclusion bodies called the Döhle bodies as seen in our patient. To date, more than 33 mutations in the MYH9 gene have been identified. However, many questions are still unanswered in the pathophysiology of non-hematological manifestations in MYH9 disorders, opening doors to new avenues of research. 

Clinical presentation

MYH9-related disorders have varied clinical presentations depending on the severity of thrombocytopenia. Bleeding (65%) is the most common symptom, especially in young females presenting with menorrhagia. Easy bruising (45%), spontaneous epistaxis (20%), and post-procedural heavy bleeding (30%) are the other presentations for these disorders. Giant platelets are present in all the affected individuals, with platelet numbers varying from 30,000/mL to 100,000/mL. On complete blood count examination, platelet histogram is indeterminate or abnormally elevated mean platelet volume serves as an indirect clue for giant platelets. Platelet aggregometry and platelet function studies using the platelet function analyzer 100 (PFA-100) are typically normal in MYH9 disorders. In MYH9 disorders, shape change in the aggregation curve is typically absent due to the altered composition of the platelet cytoskeleton; however, it is not specific to MYH9 disorders [6].

Clinical spectrum

The disease spectrum consists of four distinct diseases, which include May-Hegglin anomaly, Epstein syndrome, Fechtner syndrome, and Sebastian platelet syndrome. Myosin-9 is predominantly expressed in tissues such as blood cells, cochlea, lens, and kidneys. Hence, in addition to hematological manifestations, patients may also develop sensorineural hearing loss, renal failure, and pre-senile cataracts, as seen in our patient. The renal manifestations could range from asymptomatic nephritis to full-blown ESRD, as noticed in our patient. A review done by Tabibzadeh et al suggested the two most common histopathological renal abnormalities include firstly secondary focal segmental glomerulosclerosis with thickening and splitting of the basement membrane and focal effacement of the foot processes and secondly mesangial proliferation without immune deposits [6]. 

The following table further provides the characteristics of the four different clinical entities that comprise MYH9 disorders. According to Table 2, our patient most likely may fall under the Fechtner syndrome. 

**Table 2:** Hematological and non-hematological manifestations of MYH9 disorders As presented in a review article by Althaus and Greinacher [5] MHA: May-Hegglin anomaly, FTS - Fechtner syndrome, EPS - Epstein syndrome, SPS - Sebastian platelet syndrome

	MHA	FTS	EPS	SPS
Macrothrombocytopenia	+	+	+	+
Inclusion bodies	+	+	-	-
Hearing Loss	-	+	+	+: seen in old age
Nephritis	-	+	+	-
Cataracts	-	+	-	+: seen in old age

Management

As a hereditary autosomal dominant disorder, early detection is the key in management. Even though no specific drugs are available for these mutations, by knowing the underlying disorder, the inadvertent use of steroids, intravenous immunoglobulin (IVIG), and in some cases splenectomy can be averted. This can reduce unwarranted morbidities such as steroid-induced myopathy, diabetes mellitus, osteoporosis with fractures, cushingoid appearance, and psychosis. In addition to symptomatic management with platelet transfusions, clinical trials showing the efficacy of eltrombopag have been tested. One such study was performed in Italy in 2010 by Alessandro Pecci et al which concluded 50-75 mg of eltrombopag per day increased platelet count and reduced bleeding tendency in most patients with MYH9 disorders [7]. In another study by Alessandro Pecci, early use of angiotensin-converting enzyme improved proteinuria in MYH9 related nephritis and has slowed the progression to ESRD [8]; this emphasizes the importance of early diagnosis. Further pathophysiological mechanisms in understanding the disease process could help establish novel therapeutic agents. Another pivotal point is offering genetic counseling to family members of the affected. In our case, both the son and grandson have been educated to visit a geneticist to determine the disease entity and provide long-term counseling. 

## Conclusions

May-Hegglin disorders are often misdiagnosed as idiopathic chronic thrombocytopenic purpura (ITP) if a thorough evaluation of blood smear and a detailed bleeding history including family history is not done. Although ITP is the most common cause of unexplained thrombocytopenia, diagnosis of hereditary thrombocytopenia should be considered in the right clinical setting with family history and poor response to initial therapy. Early diagnosis of MYH9 disorders could reduce unnecessary diagnostic studies like bone marrow biopsy and even misdirected therapeutic intervention with corticosteroids, immunosuppressive agents, and splenectomy. It is important to broaden our differential and perform a comprehensive investigation of thrombocytopenia. Further research is warranted to determine new pathophysiological mechanisms and novel mutations to find suitable targets to increase the normal-sized platelet counts and reduce the giant platelets. Action must be taken before the development of irreversible conditions such as ESRD, hence early, comprehensive diagnosis of thrombocytopenia plays a life-changing role in hereditary platelet disorders. 

## References

[1] Sivapalaratnam S, Collins J, Gomez K (2017). Diagnosis of inherited bleeding disorders in the genomic era. Br J Haematol.

[2] Hussein BA, Gomez K, Kadir RA (2013). May-Hegglin anomaly and pregnancy: a systematic review. Blood Coagul Fibrinolysis.

[3] (2021). MYH9-related disease. https://ghr.nlm.nih.gov/condition/myh9-related-disorder.

[4] (2021). MYH9-related disorder. https://medlineplus.gov/genetics/condition/myh9-related-disorder/.

[5] Althaus K, Greinacher A (2009). MYH9-related platelet disorders. Semin Thromb Hemost.

[6] Tabibzadeh N, Fleury D, Labatut D (2019). MYH9-related disorders display heterogeneous kidney involvement and outcome. Clin Kidney J.

[7] Pecci A, Gresele P, Klersy C (2010). Eltrombopag for the treatment of the inherited thrombocytopenia deriving from MYH9 mutations. Blood.

[8] Pecci A, Granata A, Fiore CE, Balduini CL (2008). Renin-angiotensin system blockade is effective in reducing proteinuria of patients with progressive nephropathy caused by MYH9 mutations (Fechtner-Epstein syndrome). Nephrol Dial Transplant.

